# STOCKING THE GENETIC SUPERMARKET: REPRODUCTIVE GENETIC TECHNOLOGIES AND COLLECTIVE ACTION PROBLEMS

**DOI:** 10.1111/bioe.12098

**Published:** 2014-04-11

**Authors:** Chris Gyngell, Thomas Douglas

**Keywords:** enhancement, genetics, reproduction, collective action problems, genetic selection

## Abstract

Reproductive genetic technologies (RGTs) allow parents to decide whether their future children will have or lack certain genetic predispositions. A popular model that has been proposed for regulating access to RGTs is the ‘genetic supermarket’. In the genetic supermarket, parents are free to make decisions about which genes to select for their children with little state interference. One possible consequence of the genetic supermarket is that collective action problems will arise: if rational individuals use the genetic supermarket in isolation from one another, this may have a negative effect on society as a whole, including future generations. In this article we argue that RGTs targeting height, innate immunity, and certain cognitive traits could lead to collective action problems. We then discuss whether this risk could in principle justify state intervention in the genetic supermarket. We argue that there is a plausible prima facie case for the view that such state intervention would be justified and respond to a number of arguments that might be adduced against that view.

Various technologies already exist that enable parents to determine whether their future children will have or lack certain genetic predispositions*.* Pre-natal testing and selective abortion allow parents to decide whether to continue with a particular pregnancy based on genetic information about the developing embryo or foetus. *In vitro* fertilization (IVF) and preimplantation genetic diagnosis (PGD) allow parents to acquire genetic information about a range of embryos and then determine which to gestate on the basis of that information. In the future it may become possible for parents employing assisted reproductive technologies to decide which eggs to fertilize with which sperm on the basis of reliable genetic information about the available eggs and sperm.^1^ Advances in genetic engineering technologies could also allow parents to directly alter the genes of existing sperm, eggs, embryos or foetuses.

We use the term ‘reproductive genetic technologies’ or ‘RGTs’ to refer collectively to these technologies and to any other technologies that enable parents or others to (i) determine which of different possible future children to bring into existence based on detailed information about their likely genetic make-up, or (ii) alter the genetic make-up of a given future child whom the parents intend to bring into existence.^2^

There are at least two important questions that might be asked about RGTs. First, given the availability of specific RGTs, how ought parents to use them? That is – what are the obligations of parents with regard to using the RGTs that are available?^3^ Second, given the technical feasibility of specific RGTs, which should be made available to prospective parents? That is – how ought governments, or other regulatory bodies, to provide and regulate access to RGTs?

A popular class of responses to the second question come under the banner of ‘liberal eugenics’.^4^ Liberal eugenic approaches stress the importance of parental autonomy, and support widespread access to RGTs. One of the earliest descriptions of such an approach is found in Robert Nozick's *Anarchy, State, and Utopia*. Nozick advocates a ‘genetic supermarket’:^5^

Consider … the issue of genetic engineering. Many biologists tend to think the problem is one of design, of specifying the best types of persons so that biologists can proceed to produce them. Thus they worry over what sort(s) of person there is to be and who will control this process. They do not tend to think, perhaps because it diminishes the importance of their role, of a system in which they run a ‘genetic supermarket’, meeting the individual specifications (within certain moral limits) of prospective parents … This supermarket system has the great virtue that it involves no centralized decision fixing the future of human type(s).

The core idea of the genetic supermarket, and of liberal eugenics, is that RGTs are freely available to prospective parents, who are ultimately responsible for making selection decisions about the children. This model for regulating access to RGTs can be contrasted with older eugenic practices, which involved coercing people into certain reproductive choices, and current regulations governing access to embryo screening technologies, which in many jurisdictions restrict the use of RGTs to the prevention of diseases, or a sub-class of diseases.^6^

Most writers in the liberal eugenic tradition accept there should be some limits placed on parental use of RGTs. The genetic supermarket should not be a true free market. However, they take a true free market to be the default position, with any deviation from it requiring a justification.

There is disagreement about precisely how much deviation from a true free market is justified. Some writers argue that it is only permissible to interfere with parental choice to protect the children who are targets of these technologies.^7^ Others argue that it may also be permissible to interfere with parental choice to promote social goals like equality, and to protect the public interest.^8^

One way in which the ‘public interest’ could be harmed as a result of widespread use of RGTs is through the effect of collective action problems. We will take it that a collective action problem exists whenever rational individual agents acting in isolation from one another collectively have a negative effect on wider society.^9^

Many writers have pointed to the fact that collective action problems could potentially arise for some traits targeted by RGTs, and have claimed that this would potentially justify restricting access to these technologies.^10^ However, so far this discussion has mainly focussed on only one collective action problem: that posed by RGTs targeting height. The broader significance of collective action problems for the regulation of RGTs has not been investigated.

In this article we assess the likelihood and significance of several collective action problems that could arise in a genetic supermarket. In Part 1, we consider whether and to what extent collective action problems are likely to arise for RGTs targeting height, innate immunity and particular cognitive traits. We argue that collective action problems could arise in all three areas, with different factors affecting their extent and scope. Thus, we suggest, the concern about collective action problems is a serious one. In Part 2, we argue that if the availability of particular RGTs did result in collective action problems, then it would be appropriate for the state to restrict access to them in certain circumstances. We conclude by discussing the implications of our arguments for debates regarding the appropriate framework for regulating RGTs.

## 1. Collective Action Problems

The idea that collective action problems could potentially result from widespread access to particular RGTs has been suggested by many authors. For example, Singer says that:^11^

being able to select for height. … could start the human equivalent of the peacock's tail – an escalating ‘height race’ in which the height that distinguishes ‘tall’ people from those who are ‘normal’ increases year by year, to no one's benefit, at considerable environmental cost, and perhaps eventually even at some health cost to the children themselves. Genetic enhancement could lead to a collective action problem, in which the rational pursuit of individual self-interest makes us all worse off.

In this section we will examine the significance of the collective action problem presented by RGTs targeting height. We will also discuss two other types of RGTs that could potentially result in collective action problems that have not previously been discussed in the literature – RGTs that target innate immunity and those targeting certain cognitive traits.^12^ We assume throughout that rational parents faced with decisions about how to use RGTs would be motivated by the wellbeing of their future child.^13^ That is, if choosing between different possible future children, they would choose to have a child who can be expected to have a life containing more wellbeing over a child who can be expected to have a life containing less wellbeing, and if choosing what dispositions to bring about in a given future child, they would choose to bring about dispositions that can be expected to give the future child more rather than less wellbeing.^14^ The collective action problems we discuss arise when individual couples or single parents use RGTs in ways that are expectedly best for their children, but where parents as a collective act in a way that is bad for all of their children. No individual couple or single parent could make their future child expectedly better off by acting differently, but if all parents acted differently, all of their children would be expectedly better off.^15^

### 1.1 Height

The idea that RGTs targeting height would lead to a collective action problem builds on empirical research suggesting that tall people perform better on a range of measures thought relevant to wellbeing. Tall people have been found to be more attractive to the opposite sex and more likely to have a long-term partner.^16^ Tall people also make more money, even when factors like level of education are controlled for. Perhaps unsurprisingly, then, height has been found to be correlated with subjective wellbeing.^17^ If RGTs which target height were available in the genetic supermarket we might expect rational parents to use these RGTs to attempt to have taller children.^18^

However, if every parent used RGTs to have taller children, this would negate any positive effect of the additional height on wellbeing. This is because everyone's relative height would stay more or less the same, and it is relative height rather than absolute height that is associated with increased career and relationship success and subjective happiness. Further, there are ways in which the widespread provision of height enhancements would make everyone worse off. Buchanan and co-authors note that even if the means of height enhancement had no direct negative health consequences for the enhanced individual, such enhancements would nevertheless have costs, including the economic costs of the intervention itself and the costs of redesigning our buildings, vehicles and environment more generally to accommodate taller individuals.^19^ There may also be other environmental costs associated with height enhancements. In general tall people need to eat more food, require more fuel to travel, and consume more resources than shorter people. The creation of taller people could increase carbon emissions and increase the risk of dangerous climate change. Indeed, in a recent article, S. Matthew Liao and co-authors argue that, if we wish to use RGTs to protect our populations against climate change, we should already be aiming to make future people *shorter* than we are.^20^

Despite these costs, however, rational parents could be expected to use RGTs to have taller children. If other parents use RGTs to target height and you do not, your child will end up enduring a significant height-related disadvantage. On the other hand, if other parents do not use RGTs in this way, and you do, your child will enjoy a significant wellbeing advantage. Thus, regardless of what other parents do, if you are motivated by the wellbeing of your own future child, you will attempt to use RGTs to have taller children. The fact that choosing to have a taller child may also contribute to social and environmental costs of the sort mentioned above is unlikely to be a decisive consideration for parents motivated solely by the wellbeing of their future child, since most of those costs will be borne by others. In theory then, the availability of RGTs which target height in the genetic supermarket would create a collective action problem. The rational actions of individuals in the market would make everyone worse off.

However the problem may not be as significant as it is sometimes presented. For one, the relationship between height and subjective wellbeing is strongly affected by sex. Only in males is height independently correlated with increases in wellbeing, once economic and health impacts are controlled for.^21^ Taller women are happier only because they are, on average, healthier and earn more money. The association between height and wellbeing in women may therefore be better explained by factors which cause both tallness and elevated wellbeing rather than a direct relationship between height and wellbeing. Malnourishment in childhood, for example, may lead to individuals being shorter as adults, and may also prevent them from reaching their full cognitive potential. This could contribute to worse educational outcomes and earnings in adulthood.^22^ Consequently, we might expect that direct height enhancements would be more popular among parents of male children than female children. Further, at very extreme heights it is doubtful whether further height increases will be associated with increases in wellbeing. Being extremely tall has health and social costs. At extreme heights individuals can find it difficult to attract romantic partners.^23^ Being very tall can lead to cardiovascular problems, because of the increased load on the heart to supply the body with blood. It can also lead to problems resulting from the increased time it takes the brain to communicate with the extremities. If humans were to get taller and taller, at some point any relative height advantage would surely be outweighed by these costs.

Therefore, while widespread access to height enhancements will potentially lead to a collective action problem, this problem might be somewhat limited in scope (due to the fact that height does not appear to confer a wellbeing advantage on women) and in magnitude (due to the fact that increasing height is likely to cause a net loss of wellbeing at some point).

### 1.2 Innate immunity

The widespread availability of RGTs capable of targeting innate immunity could also lead to collective action problems. In a genetic supermarket some immune system genes may be more desirable than others, as they provide protection against the likeliest disease threats. However, if many parents pick the same immune system genes for their children, their combined actions may reduce population level immunodiversity, and this could make everyone worse off.

Some genes provide protection against some diseases but increase susceptibility to others. For example, it is known that a variant of the DARC gene – which codes for an antigen found on red blood cells – provides protection against malaria. However this version of the gene also disposes people to be more susceptible to human immunodeficiency virus (HIV).^24^ Genes like this could potentially lead to collective action problems, as it may be best for any given individual to have one variant, but best for the overall wellbeing of the population for there to be a mixture of the two variants in the population. For example, imagine a population in which the average incidence of malaria is 3% and the incidence of HIV is 5%, and assume that it is at least as bad to have HIV as to have malaria. If everyone chose the version of the gene that was expectably best for their children, they would pick the version of the gene which provided protection against HIV. However this could make the population as a whole worse off. If a mixture of genes were present in the population, this might ensure that any epidemics of HIV or malaria would be only moderately severe. However, if everyone had the gene that protects against HIV and leaves them susceptible to the malaria virus, the result could be that malaria epidemics would tend to be very severe. Even if there were a corresponding reduction in the severity of HIV epidemics, this could be a negative outcome overall. Severe epidemics may be particularly undesirable as they result in many people being sick simultaneously, which can disrupt the supply of essential goods and services. This can lead to worse outcomes for those directly affected by an illness as well as the broader population. For example, as the supply of health services can be disrupted, sick individuals may have trouble getting properly diagnosed and treated. Similarly, as the supply of other essential goods can be disrupted, severe epidemics can have negative flow-on effects for healthy individuals. Therefore, in cases such as these, it may be preferable for a population to maintain a certain amount of genetic diversity. Diversity would reduce the likelihood that a significant portion of the population would become sick at the one time.

Other immune genes have known benefits but may also have costs that are yet to be discovered. These genes could also pose collective action problems if available in a genetic supermarket. For example, the CCR5 gene codes for a type of receptor found on macrophages (a type of white blood cell), which are targeted by the HIV virus. One form of the CCR5 gene provides resistance to the HIV virus.^25^ However, given the important role played by macrophage receptors in fighting other infections, it is possible that individuals with this form of the gene will be more susceptible to other infectious agents that are yet to evolve. If this gene were available in a genetic supermarket it seems plausible that many parents would select the form of the gene which provides resistance to HIV. This is likely to be the case even in populations where HIV is only a minor threat. But the combined result of many people selecting this gene for their children may be bad for those populations as a whole, as it may increase their susceptibility to future epidemics.

This problem could be exacerbated if many different immune genes could be targeted by RGTs. If parents make many similar decisions across a range of immune genes, a significant reduction in the general immune-diversity of a population may result. This general reduction in genetic diversity could make these populations prone to being devastated, and even wiped out, by novel disease threats.

Of course, it might be thought that, while in theory RGTs that target immunity will result in collective action problems, the ability of a human population to fight disease in other ways may render these problems insignificant by the time a genetic supermarket opens. Vaccines and antibiotics can already mitigate many infectious disease threats, and in the future other technologies may make innate immunity even less important than it is now. However, it is difficult to be confident that innate immunity will be less important in the future. Antibiotic resistance is becoming a major issue and if it continues we may even find that innate immunity will be more important for the population than it is now.

### 1.3 Cognitive traits

RGTs targeting certain cognitive traits could also lead to collective action problems.^26^ Some cognition-related genes may be very popular in an unregulated genetic supermarket. However, the combined action of many parents choosing these genes for their children may reduce valuable types of cognitive diversity and make everyone worse off.

Recent work in social science has demonstrated that when groups of people are solving complex problems, cognitive diversity can matter more than individual ability.^27^ Cognitive diversity in this sense refers to differences in how each ‘individual sees the world, interprets its problems, and makes predictions in it’.^28^ Groups with low levels of cognitive diversity tend to get stuck on sub-optimal solutions when attempting to solve complex problems together. Because individuals in these groups have similar ways of approaching the problem, they will not be able to see the whole range of potential solutions available. In contrast, when cognitively diverse groups are solving problems together, they can assess more potential solutions, meaning they are more likely to find optimal solutions to problems.^29^ This model is supported by data showing that cognitively diverse teams outperform less diverse teams on measures of problem solving.^30^

If this model is accurate, cognitive diversity may influence the collective wellbeing of a society. Cognitive diversity makes groups of people better at solving problems, and populations benefit from this. Reducing cognitive diversity could potentially have very significant long-term effects on future generations, as it could diminish society's ability to deal with complex global problems like climate change.

In many circumstances we would not expect all rational parents to pick the same cognition targeted genes for their children, and so cognitive diversity might not be significantly reduced by the availability of RGTs that affect cognition. Generally, rational individuals have many diverse preferences, and so we may expect them to make diverse choices regarding which cognition-affecting genes they pick for their children. However some preferences may be very widely shared by many parents. For instance, it seems plausible that most parents want their children to be happy. Studies indicate that parents generally prefer teachers that make their children happy over ones that increase their academic performance.^31^ Therefore, if some genes make it more likely children will be happy we may expect many rational parents to select them for their children. If this has the effect of lowering a valuable type of cognitive diversity, it may make everyone worse off.

Consider genes that predispose individuals to depression. Being prone to depression can make someone's life harder and less enjoyable. This may mean that, in a genetic supermarket, rational individuals would select against genes that predispose to depression. However, these genes may also contribute to valuable cognitive skills. For example, people who are predisposed to depression have been shown to have increased analytic skills.^32^ Research also suggests that people who are depressed use different heuristics to solve problems from those used by people who are not depressed.^33^ The existence of individuals on the depressive spectrum, then, could constitute a valuable type of cognitive diversity – one that contributes to collective wellbeing.^34^

Another example of a cognitive trait that may influence happiness is extroversion. A variety of studies have linked being extroverted to increased levels of subjective wellbeing.^35^ This means that if RGTs were available which targeted extroversion, we may expect rational parents who value happiness for their children to take steps to increase their chance of having an extroverted child. If many parents did this, it would have the effect of reducing the population level diversity of this trait. But this could also be bad for the population as a whole. Studies indicate that introverts and extroverts have differences in brain structure,^36^ and respond differently to stimuli.^37^ It's plausible that this contributes to distinctive perspectives and heuristics and represents a valuable type of cognitive diversity.

The widespread availability of RGTs targeting extroversion and depression may, therefore, pose a collective action problem. It may be rational for parents who value happiness to use the RGTs to select against genes that predispose individuals to depression and introversion. But this could be detrimental for the population as a whole because it reduces a valuable kind of cognitive diversity.

## 2. Collective Action Problems and the Role of the State

In the previous section, we looked at some collective action problems that may arise in a genetic supermarket. We argued that the availability of RGTs that target height, immunity and aspects of cognition could result in collective action problems. At least in the case of immunity and cognitive traits, and possibly also in the case of height, it is plausible that these problems would have significant effects on society and future generations, though of course any predictions about the likely scope and extent of such problems are necessarily highly uncertain.

In this section we will focus on ethical and political issues rather than empirical ones. If a particular RGT did lead to a collective action problem, should that influence whether the state restricts access to it?

Some would argue that there is a strong case for state interference in a market to prevent collective action problems. The presence of a collective action problem can be seen as a type of market failure, which some take to be a ground for state intervention.^38^ This suggests that some would think the state could be justified in intervening in a genetic supermarket in order to prevent collective action problems. This view is prima facie quite plausible. It is plausible that moral agents, including the state, have moral reasons to promote and not to set back human wellbeing, and collective action problems of the sort that we have discussed would tend to reduce overall human wellbeing. On this view, then, the state would have moral reasons to prevent those problems from occurring.

However some influential moral views imply that these reasons are illusory or at least are outweighed by other considerations. It may be claimed either that no state involvement at all in the genetic supermarket is appropriate, or that the state should only intervene in the market to prevent imminent and substantial harm to individuals. We consider these views in turn below.

### 2.1 A laissez faire approach

Perhaps the genetic supermarket should be a true ‘free market’ to which no state restrictions apply. The most promising argument for this view would, we think, appeal to the view that parents have a *right* to determine the genetic characteristics of their children. This view might (though need not) be advanced within an entitlement-based theory of justice such as that advanced by Robert Nozick.^39^ If parents enjoy a right of this kind, then state intervention in the genetic supermarket would be impermissible, or at least presumptively impermissible, depending on what view one takes regarding the normative strength of rights.

A difficulty with this approach, however, is that it is very doubtful whether parents do enjoy a right of the relevant sort. It is often claimed that people have a right to self-ownership and it might, perhaps, follow from this that they have a right to determine *their own* genetic characteristics, insofar as this is possible. But it is doubtful whether this right extends to one's children who are not part of one's own self. Alternatively it might be claimed that parents have rights to determine the genetic characteristics of their offspring because children are the external property of their parents, and parents have a right to shape the characteristics of their external property. But children do not seem to fall under property rights of the sort that cover external possessions; we do not, for example, think that parents are free to sell, rent or destroy their children as we would were children external property of the ordinary sort. It seems doubtful, then, that an appeal to property rights could support a right to determine the genetic characteristics of one's children.

Rather than that attempting to derive a right to determine the genetic characteristics of one's children from a more general class of property rights, one might attempt to derive it from a more general right to shape the characteristics of one's children. It is true that parents are normally permitted to exert considerable influence over the traits of their children by, for example, choosing what sort of parenting style to adopt and choosing what kinds of educational and recreational opportunities to present to their children. It might be argued that we allow parents such great freedom in these areas because we take them to have a right to shape the traits of their child, and this right might be thought to include a right to determine, prior to birth, the child's genetic characteristics.

However, most people would accept that there should be significant constraints on how parents raise their children. For example, compulsory elementary education is widely accepted, so is the idea that the state may intervene with parental freedom in cases of child neglect or where parents are, for example, encouraging seriously anti-social behaviour in their children. Insofar as widely held views on parenting support a right to determine the characteristics of one's children, and thus to determine the genetic characteristics of one's future children, they support only a rather constrained right. It is therefore difficult to see how an appeal to such views could support an *unconstrained* right of the sort that would be necessary to support an unregulated genetic supermarket approach to RGTs.

### 2.2 The ‘real and present’ harms requirement

Rather than a true free market, perhaps the genetic supermarket should be one in which the standard for state intervention is set very high, so that it precludes interference to prevent collective action problems, though it may allow state intervention for other reasons. John Harris suggests a view that might have this implication. He states that, for restrictions on access to reproductive technologies to be justified, it must be the case that the use of those technologies would be

seriously harmful to others or to society and that these harms are real and present, not future and speculative, for if they were not, the presumption in favour of liberty would be at risk whenever imaginative tyrants could postulate possible, but highly unlikely, future harms.^40^

It might be argued that the sorts of harms caused by creating collective action problems do not satisfy this requirement. However, it is unclear what Harris means by his requirement that harms be ‘*real and present, not future and speculative*’.^41^ The requirement may seem to rule out, as grounds for state intervention, all harms that will not occur immediately, or at least in the short-term future. But if so, the requirement will be implausible. In general, the fact that a harm will occur some distance into the future does not undermine the case for state intervention. For example, a government could clearly be justified in preventing a parent from feeding a poison to his child, even if that poison would only cause harm to the child years down the line.

This suggests that we should focus not on Harris's distinction between ‘present’ and ‘future’ harms, but on his distinction between ‘real’ and ‘speculative’ harms. There are different ways in which we might interpret this distinction. On one interpretation, a harm qualifies as ‘real’ if and only if there is some *non-negligible* probability of it occurring: entirely fantastic harms do not warrant state intervention, but harms could nevertheless be highly unlikely and still warrant government intervention. This view would certainly not rule out collective action problems of the kinds we have described as grounds for state intervention, since the probability that such problems will occur is not negligible. However, it is doubtful that Harris intends to set the threshold for ‘real’ harms so low, for he describes the ‘real and present harm’ condition as a ‘high standard’.^42^ He also at times replaces talk of ‘real and present’ harms with talk of ‘real, present, and highly probable’ harms.^43^

This suggests that Harris rather has in mind that a harm would qualify as ‘real’ only if there is a *high* probability that the harm will occur. Harris would then be claiming that only harms with a high probability justify state intervention. This might seem to exclude consideration of the sorts of harms that we have discussed. However, on this interpretation, Harris view is implausible. Surely states are permitted to intervene to prevent harms that will occur with low probability if those harms are severe enough. Suppose parents feed their child a poison that only has an effect in 1% of cases, but that effect is to cause death. The state would be permitted to intervene to prevent the administration of this poison.

Perhaps Harris's ‘real harm’ requirement could be understood in a different and more plausible way. The thought might be that a harm will be sufficient to justify state intervention only if it will occur with a high probability *or* would have a high severity. On this view, low probability harms may justify state intervention, but only if they have a high degree of severity. This criterion would seem to preserve Harris' thought that there should be a presumption in favour of liberty. However, it is not clear that this criterion would rule out intervention in the genetic supermarket to prevent collective action problems. After all, it is plausible that, for example, uses of RGTs that significantly reduced diversity of cognitive traits or immunity *would*, with at least some significant probability, cause very severe harms. A reduction in immune system diversity might, for example, result in a serious and lethal epidemic that would kill thousands of people.

Of course, it might be argued that no individual parent's choice to use an RGT would have made much difference to the risk that such an epidemic would occur. No individual parent would have created a significant risk of a serious harm. However, *collectively* the parents whose choices did in fact make it occur *would* have contributed to a risk of a very serious harm, and it is difficult to see why collective actions should be immune to the kind of state intervention that might be justified in relation to individual actions which risk serious harm. Imagine a case like Parfit's ‘harmless torturers’, in which each of a 1000 individuals pushes a button and the 1000 button-presses *together* result in one instance of torture.^44^ Although no one individual makes a perceptable difference to pain experienced by the victim in Parfit's thought experiment, it would surely be permissible for a government to intervene to prevent some (or perhaps all) of the 1000 people from pressing the button. More generally, if the actions of groups of individuals only *together* constitute a significant risk of serious harm, it may still be permissible to restrict the actions of each individual. Many environmental regulations, for example, such as those which prohibit the burning of household waste or the use of inefficient fuels, are intended to prevent (risks of) environmental harms that would only be severe if many people engaged in the actions in question. Yet most would accept that these regulations can be justified.

Are there other grounds, besides those mentioned by Harris, on which one could argue that the harms produced by collective action problems would be insufficient to justify state interference in the genetic supermarket?

One suggestion might be that the harms produced by collective action problems are insufficient because they are *noncomparative* harms.^45^ The collective action problems we have discussed may cause significant harms some way into the future, but genetic decisions that produce those harms will also affect what people come into existence in the future. Suppose a large number of people choose to have children with genes that protect against HIV, but this leaves future people susceptible to some new and highly lethal infectious disease 200 years from now, such that no-one who will actually exist would also have existed if the genetic selection decisions had not been made. But suppose those genetic selection decisions will also influence what people exist 200 years from now. In that case, arguably no-one would suffer what Jeff McMahan would call a *comparative* harm – no-one would be made worse off than they would otherwise have been, or than they were previously, by the occurrence of the epidemic, since the people that it afflicts would not have existed had the decisions that caused the epidemic not been made. At most, we could say that those afflicted by the epidemic suffer a noncomparative harm – the sort of harm that exists whenever one experiences suffering, regardless of whether things would have been better for the individual otherwise, or were better previously.

This suggestion seems unpromising however. For one thing, it is not clear that noncomparative harms are insufficient to justify state intervention. Derek Parfit gives the example of a community which must choose between *risky policy* – which would make inhabitants of the community slightly better for the next century but cause a catastrophe in three centuries; and *safe policy* – which would avoid the catastrophe but make inhabitants slightly worse off over the next century.^46^ The choice in policy would also change which people exist in the future, so that none of the individuals who would be affected by the catastrophe in risky policy would exist if the community adopted safe policy. By contrast, some of the individuals who will exist in the next century if *safe policy* is adopted would have existed, and been better off, had that policy not been adopted. Despite the fact that *risky policy* would only result in noncomparative harms, while safe policy would result in some comparative harms, it is intuitively plausible that that a state would be justified in choosing *safe policy*. Similarly we may think that states are justified in implementing measures that mitigate the long term risks of climate change, even if these policies also change which people exist in the future, and therefore only prevent noncomparative harms.

Further, it is not clear that all harms produced by the collective action problems we have discussed would be noncomparative. Suppose that current selection decisions regarding immune system or cognitive traits had the effect of causing future epidemics or slowing the rate of future scientific progress. Unless those effects took a very long time to become manifest, some of the people who experience them will be people who already exist. Those people would be made (in one way) worse off by these effects than they would otherwise have been. They would suffer *comparative* harms.

Another suggestion might be that the harms produced by collective action problems would not justify state intervention in the genetic supermarket because they would not affect currently identifiable individuals. In general people have a tendency to give more weight to the claims of identified individuals than unidentified individuals. For example, people are more likely to voluntarily contribute money to rescue an identified group of trapped miners, than they are to voluntarily contribute money to improve mine safety, which would prevent more miners whose identities we do not yet know, becoming tapped in the future.^47^ However whether this general tendency reflects a morally important distinction is disputed.^48^ In many cases it seems that harms affecting identified and unidentified individuals should be considered as equally important. For example, we think the police should go to the same lengths to catch a criminal who is planning on killing a specific individual as they do in catching one planning on killing a random person.

But even if it were true that states should give harms affecting identified individuals greater weight than harms affecting unidentified individuals, it does not follow that no measures should be taken to prevent collective action problems in a genetic supermarket. This is because it is implausible that harms affecting unidentified individuals should carry *no* weight at all. If the only way to prevent the deaths of many unidentified individuals in an armed attack was to restrict the freedom of one individual to purchase automated weapons, this would be clearly justified. Similarly if the risk of harm posed by collective action problems to unidentified individuals is sufficiently large, the state would surely be justified in imposing some restrictions on parents accessing the genetic supermarket in order to prevent them.

## 3. Conclusion

In this article we have investigated the significance of some possible collective action problems that could result from widespread access to RGTs. We claimed that such problems could be expected to arise from the free availability of RGTs targeting height, immune system traits and certain cognitive capacities. We then examined whether the risk of these problems could in principle justify state intervention in a genetic supermarket. First, we noted that there is a plausible *prima facie* case for the view that such interventions could be justified, and second, we responded to a number of arguments that might be adduced against that view. We do not claim to have provided knock-down objections to each of these arguments. However, we hope that our discussion has cast significant doubt on them. It is possible that these doubts could be overcome. However, we believe that, in the meantime, it is reasonable to believe that concerns about collective action problems could in principle justify regulation of the genetic supermarket. We say ‘in principle’ because we may never have strong enough evidence that a particular collective action problem will occur, or will be serious enough, to warrant government intervention. We have noted several areas in which it is *plausible* that serious collective actions would occur, but that is not to say that we have, or will have, robust and specific evidence of the sort that might be necessary to justify government intervention in relation to particular RGTs.^49^

As stated in the introduction, there is disagreement among proponents of liberal eugenics about exactly what conditions justify state interference in the genetic supermarket. Ronald Bailey argues that ‘to the extent that new biotechnologies need regulation, agencies should be limited to deciding, as they have traditionally done, only questions about safety and efficacy.’^50^ Bailey rejects the notion that the state should intervene in the genetic supermarket for reasons beyond the protection of individuals. John Harris accepts that the state could intervene in the genetic supermarket for social reasons, but only to prevent ‘real and present harms or dangers’; he takes this to be a ‘high standard’.^51^

Jonathon Glover is less restrictive in his requirements for state interference in the genetic supermarket. Glover writes:

could leaving people free to choose genes for their children at the genetic supermarket have serious social costs? If so, we may need a regulated market, on a European model. On this system, there would be no state plan to change people's genes or to improve the gene pool, but there might be limitations on genetic choices thought to be against the public interest.^52^

Allen Buchannan and co-authors endorse a similar position when they say ‘society has good, if not conclusive, reason to restrict the liberties of individuals if the exercise of those liberties undermines a public good’.^53^

We believe the arguments presented in this article support a framework for regulating RGTs that is more in line with those proposed by Buchannan and Glover than those proposed by Harris and Bailey. We have argued that, in principle, it is legitimate for the state to intervene in the genetic supermarket to prevent collective action problems. The types of harms that collective action problems pose may not affect any identifiable individuals and may not occur for several generations, meaning they may not be considered legitimate grounds of state interferences in more restrictive liberal eugenic approaches. Nevertheless, given the total risk of harm that may be posed by collective action problems, we believe they would potentially justify restrictions on parental choice in the genetic supermarket.

## Footnotes

1 This possibility seems likely to occur in the very near future. Personal genomics Company ‘23andMe’ recently received a patent to a technology called ‘Gamete Donor Selection Based on Genetic Calculations’ (US patent: us 8,543,339). Technologies such as this would allow individuals to choose between sperm or egg donors based on the statistical likelihood of the resulting child having a certain phonotype.

2 We understand ‘child’ to mean ‘post-natal child’. Thus, for us, a child comes into existence at the time of its birth. We suspect that much of what we say would also bear on practices that influence the capacities or genetic material of existing children and adults, but we do not discuss such practices here.

3 Various principles have been offered in response to this first question. See, for example, J. Robertson. *Children of Choice: Freedom and the New Reproductive Technologies*. Princeton, NJ: Princeton University Press, 1994; S. Shiffrin. Wrongful life, procreative responsibility, and the significance of harm. *Legal Theory* 1999; 5: 117–148; J. Savulescu. Procreative beneficence: Why we should select the best children. *Bioethics* 2001; 15: 413–426; E. Harman. Can we harm and benefit in creating? *Philosophical Perspectives* 2004; 18: 89–113; J. Elster. Procreative beneficence – Cui bono? *Bioethics* 2011; 25: 482–488; T. Douglas & K. Devolder. Procreative altruism: Beyond individualism in reproductive selection. *J Med Philos* 2013; 38: 400–419.

4 See, for example, N. Agar. Liberal Eugenics. *Public Aff Q* 1998; 12: 137–155; A Buchanan et al. *From Chance to Choice: Genetics and Justice*. Cambridge: Cambridge University Press; 2001; N. Agar. *Liberal Eugenics: In Defence of Human Enhancement*. Malden, MA: Blackwell Publishing; 2004; J. Harris. *Enhancing evolution: the ethical case for making better people*. Princeton, N.J: Princeton University Press; 2007; J. Glover. *Choosing Children: Genes, Disability, and Design*. Oxford: Oxford University Press; 2008; P. Singer. Parental Choice and Human Improvement. In: *Human Enhancement*. J. Savulescu, N. Bostrom, eds. Oxford: Oxford University Press; 2009. pp. 277–289.

5 R. Nozick. *Anarchy, State, and Utopia*. New York: Basic Books; 1974. p. 315.

6 Another practice that is often allowed under current regulations is the selection of embryos on the basis of their suitability to act as a tissue donor for a sick sibling. Children who are born through this process are often referred to as ‘saviour siblings’. For discussion, see M. Spriggs & J. Savulescu. Saviour siblings. *J Med Ethics* 2002; 28(5): 289–289. We thank an anonymous reviewer for this point.

7 R. Bailey. *Liberation Biology: *The Scientific and Moral Case for the Biotech Revolution**. Amherst: Prometheus Books; 2005.

8 Buchanan et al., *op. cit.* note 4, pp. 16–17 & 183. Glover, *op. cit.* note 4, pp. 77–79.

9 We understand ‘wider society’ to include both current and present individuals, and both individuals who contribute to the problem and those who do not. Economic definitions of collective action problems are frequently somewhat narrower than ours, but we believe that our definition captures how the concept has been used in discussions of RGTs.

10 Buchanan et al., *op. cit.* note 4, pp. 182–187; Singer, *op. cit.* note 4.

11 Singer, *op. cit.* note 4, pp. 282–283.

12 Widespread access to sex selection technologies may also result in collective action problems in certain parts of the world. If it is in an individual's best interest to be a particular sex because of certain social conditions, the widespread availability RGTs targeting sex may make everyone worse off by causing a large imbalance in the sex ratio. We have chosen not to discuss this particular collective action problem in detail because the likelihood that it will arise and its significance if it does arise differ substantially between different societies. For further discussion see Buchanan et al., *op. cit*. note 4, pp. 183–184; and R. Sparrow. Human enhancement and sexual dimorphism. *Bioethics.* 2012; 26: 464–475.

13 In this context we use the term ‘future child’ to refer to the next child the parents will have, even if the parents are using technologies which change the identity of that child, such as pre-implantation genetic diagnosis.

14 We thus effectively assume that rational parents would *maximize* the wellbeing of their children given the possibilities open to them. However, our subsequent arguments would not be affected if instead they would only aim for a high, but not necessarily maximal, level of wellbeing.

15 Although all the collective action problems we discuss are ones in which parents act in ways that are expectedly best for their children, in some cases parents may use RGTs in ways that actually end up making their children individually worse off because things do not turn out as expected. The collection action problem arising from RGT's targeting innate immunity, discussed below, takes this form.

16 D. Nettle. Women's height, reproductive success and the evolution of sexual dimorphism in modern humans. *Proc Biol Sci* 2002; 269: 1919–1923.

17 A. Deaton & R. Arora. Life at the top: The benefits of height. *Econ Hum Biol* 2009; 7: 133–136.

18 Of course, insofar as parents are limited to selecting between embryos created using existing in vitro fertilization techniques, they will be severely constrained in the extent to which they can influence the height, or other traits, of their offspring. This is because these techniques allow for the creation of only a small number of embryos. However, it is possible that these constraints will be weakened in the future through the development of genetic modification techniques or stem cell techniques that would enable the creation of eggs and sperm in vitro. For discussion of the latter possibility, see H. Bourne, T. Douglas & J. Savulescu. Procreative Beneficence and in Vitro Gametogenesis. *Monash Bioeth Rev* 2012; 30: 29–48; and R. Sparrow. In Vitro Eugenics. *J Med Ethics.* Forthcoming.

19 Buchanan et al., *op. cit.* note 4, p. 186.

20 S.M. Liao et al. Human Engineering and Climate Change. *Eth Pol Envir* 2012; 15: 206–221.

21 V. Carrieri & M. De Paola. Height and subjective well-being in Italy. *Econ Hum Biol* 2012; 10: 289–298.

22 A. Case & C. Paxson. Stature and Status: Height, Ability, and Labor Market Outcomes. *J Poliy Econ* 2008; 116(3): 499–532.

23 Nettle, *op. cit.* note 16.

24 W. He et al. Duffy antigen receptor for chemokines mediates trans-infection of HIV-1 from red blood cells to target cells and affects HIV-AIDS susceptibility. *Cell Host Microbe* 2008; 4: 52–62.

25 M. Samson et al. Resistance to HIV-1 infection in caucasian individuals bearing mutant alleles of the CCR-5 chemokine receptor gene. *Nature* 1996; 382: 722–725.

26 Many cognitive traits are significantly influenced by genetic factors. For example, degree of extroversion and risk of depression both have high heritability, indicating that a significant amount of the variation observed in these traits can be explained by genetic differences (see T.J. Bouchard, M. McGue. Genetic and environmental influences on human psychological differences. *J Neurobiol* 2003; 54: 4–45). This suggests a range of cognitive traits could potentially be targeted by RGTs.

27 L. Hong & S.E. Page. Groups of diverse problem solvers can outperform groups of high-ability problem solvers. *Proc Natl Acad Sci USA* 2004; 101: 16385–16389; S.E. Page. *The Difference: How the Power of Diversity Creates Better Groups, Firms, Schools, and Societies*. Princeton: Princeton University Press; 2008.

28 H. Landemore. Deliberation, cognitive diversity, and democratic inclusiveness: an epistemic argument for the random selection of representatives. *Synthese* 2013; 190: 1209–1231.

29 How much cognitive diversity is beneficial for a particular group will depend on the problem the group is trying to solve. For simple problems only very low levels of cognitive diversity may be needed. For more complex problems groups will benefit from much higher levels of cognitive diversity (although there will still be an upper limit above which extra diversity is no longer beneficial). See Page, *op cit.* note 27, pp. 158–159.

30 Page, *op cit.* note 27; L.L. Martins et al. A Contingency View of the Effects of Cognitive Diversity on Team Performance: The Moderating Roles of Team Psychological Safety and Relationship Conflict. *Small Gr Res* 2013; 44: 96–126.

31 B.A. Jacob & L. Lefgren. What Do Parents Value in Education? An Empirical Investigation of Parents' Revealed Preferences for Teachers. *Q J Econ* 2007; 122: 1603–1637.

32 P.W. Andrews & J.A. Thomson Jr. The bright side of being blue: depression as an adaptation for analyzing complex problems. *Psychol Rev* 2009; 116: 620–654.

33 R. Greifeneder & H. Bless. Depression and reliance on ease-of-retrieval experiences. *Eur J Soc Psychol* 2008; 38: 213–230.

34 It should be noted that the arguments presented here could also apply to other treatments for depression, such as the use of antidepressants. These could also reduce the types of cognitive diversity discussed here and thus result in collective action problems. We thank an anonymous reviewer for suggesting this possibility.

35 C.R. Gale et al. Neuroticism and Extraversion in Youth Predict Mental Wellbeing and Life Satisfaction 40 Years Later. *J Res Pers* 2013; 47: 687–697.

36 D.W. Johnson et al. Cerebral Blood Flow and Personality: A Positron Emission Tomography Study. *Am J Psychiatry* 1999; 156: 252–257.

37 L.D. Smillie et al. Do extraverts get more bang for the buck? Refining the affective-reactivity hypothesis of extraversion. *J Pers Soc Psychol* 2012; 103: 306–326.

38 For example see N. Stern. The Stern Review on the Economic Effects of Climate Change. *Popul Dev Rev* 2006; 32: 793–798.

39 Nozick, *op. cit.* note 5.

40 Harris, *op. cit.* note 4, p. 74.

41 We understand the category of harms to include both comparative harms and noncomparative harms (see, for discussion of this distinction, J. McMahan. Causing People to Exist and Saving People's Lives. *J Ethics* 2013; 17: 5–35). A person suffers a comparative harm when she is made worse off than she was previously, or than she would otherwise have been. A person suffers a noncomparative harm when she exists in a bad state, and irrespective of how well of she was previously, or would have been in counterfactual situations. We return to this distinction later in this section.

42 Harris, *op. cit.* note 4, p. 72.

43 See for example ibid: 80.

44 D. Parfit. *Reasons and Persons*. Oxford: Oxford University Press; 1987. p. 79.

45 McMahan, *op. cit.* note 41.

46 Parfit, *op. cit.* note 44, p. 171.

47 K. Jenni & G. Loewenstein. Explaining the Identifiable Victim Effect. *J Risk Uncertain* 1997; 14: 235–257.

48 N. Daniels. Reasonable Disagreement about Identifed vs. Statistical Victims. *Hastings Cent Rep* 2012; 42: 35–45.

49 In some cases the best way to determine whether access to a particular RGT will result in a collective action problem may be to make it widely available and monitor its use. If it is observed that a significant number of people are using the RGT in the sorts of ways that give rise to collective action problems, access to it could then be restricted. We thank an anonymous reviewer for suggesting this possibility.

50 Bailey, *op. cit*. note 7, p. 235.

51 Harris, *op. cit.* note 4, pp. 79, 72.

52 Glover, *op. cit*. note 4, p. 77.

53 Buchanan et al., *op. cit.* note 4, p. 183.

